# Four putative pathogenic *ARHGAP29* variants in patients with non-syndromic orofacial clefts (NsOFC)

**DOI:** 10.1038/s41431-024-01727-3

**Published:** 2024-11-06

**Authors:** Peyman Ranji, Eleonore Pairet, Raphael Helaers, Bénédicte Bayet, Alexander Gerdom, Vera Lúcia Gil-da-Silva-Lopes, Nicole Revencu, Miikka Vikkula

**Affiliations:** 1 Human Molecular Genetics, de Duve Institute, University of Louvain, Brussels, Belgium; 2 Centre Labio-Palatin, Division of Plastic Surgery, Cliniques universitaires Saint-Luc, University of Louvain, Brussels, Belgium; 3 Department of Translational Medicine, Area of Medical Genetics and Genomic Medicine, University of Campinas (UNICAMP), Campinas, SP Brazil; 4 Center for Human Genetics, Cliniques universitaires Saint-Luc, University of Louvain, Brussels, Belgium; 5 WELBIO Department, WEL Research Institute, avenue Pasteur, 6, 1300 Wavre, Belgium

**Keywords:** DNA sequencing, Mutation, Genotype, Genetic counselling

## Abstract

The pathophysiological basis of non-syndromic orofacial cleft (NsOFC) is still largely unclear. However, exome sequencing (ES) has led to identify several causative genes, often with reduced penetrance. Among these, the Rho GTPase activating protein 29 (*ARHGAP29*) has been previously implicated in 7 families with NsOFC. We investigated a cohort of 224 NsOFCs for which no genetic pathogenic variant had been identified by diagnostic testing. We used ES and bioinformatic variant filtering and identified four novel putative pathogenic variants in *ARHGAP29* in four families. One was a missense variant leading to the substitution of the first methionine with threonine, two were heterozygous frameshift variants leading to a premature termination codon, and one was a nonsense variant. All variants were predicted to result in loss of function, either through mRNA decay, truncated ARHGAP29, or abnormal N-terminal initiation of translation of ARHGAP29. The truncated ARHGAP29 proteins would lack the important RhoGAP domain. The variants were either absent or rare in the control population databases, and the loss of intolerance score (pLI) of ARHGAP29 is 1.0, suggesting that ARHGAP29 haploinsufficiency is not tolerated. Phenotypes ranged from microform cleft lip (CL) to complete bilateral cleft lip and palate (CLP), with one unaffected mutation carrier. These results extend the mutational spectrum of *ARHGAP29* and show that it is an important gene underlying variable NsOFC phenotypes. *ARHGAP29* should be included in diagnostic genetic testing for NsOFC, especially familial cases, as it may be mutated in ∼4% of them (4/97 in our cohort) with high penetrance (89%).

## Introduction

Orofacial clefts, such as Cleft lip with or without cleft palate (CL/P), and Cleft palate (CP), are one of the most common craniofacial birth defects, with an incidence of 1 in 700–1000 live births [1]. They are divided into syndromic orofacial cleft (SyOFC) and non-syndromic orofacial cleft (NsOFC) groups depending on whether the patient has other anomalies or not. Approximately 70% are NsOFC patients, of which 80% are sporadic and around 20% are familial cases [1, 2]. SyOFC is often inherited according to Mendelian inheritance, while NsOFC has a complex multifactorial etiology causing variable phenotypes and penetrance.

A number of approaches have been used to identify potential genetic factors underlying NsOFC, including linkage studies, genome-wide association studies (GWAS) and, more recently, exome sequencing (ES). GWAS studies could identify over 40 loci which may account for about 30% of the heritability of NsOFC [3–5]. Some part of the missing heritability could be due to rare variants in NsOFC patients that were not detected by GWAS. It has been shown that variants in the genes generally known to cause SyOFC can be identified in about 10% of NsOFC cases [6]. Overall, gaps in our knowledge of the genetic basis of NsOFC need to be addressed to enhance genetic counseling, improve risk prediction, and guide research efforts.

Several genes have been identified as causative for NsOFC. One of them is *ARHGAP29* [7, 8]. It encodes the Rho GTPase activating protein (GAP) 29, a GTPase activator, which converts Rho-type GTPases to an inactive GDP-bound state [9]. *ARHGAP29* suppresses RhoA signaling and attenuates the activity of *ROCK* and *MYH9* in endothelial, mesenchymal, and oral epithelial cells, as well as keratinocytes [9–11]. Rho signaling has a crucial function in cell shape, movement, cell-cell interactions, and proliferation in various cellular processes, such as craniofacial development [9, 12].

GWAS studies followed by Sanger sequencing or targeted NGS identified 25 deleterious variants in *ARHGAP29* in NsOFC patients, suggesting the role of this gene is NsOFC pathogenesis, and as an etiological factor at the 1p22 locus [7, 13–18]. Eight of the 25 variants can be considered likely pathogenic based on pathogenicity prediction algorithm(s) and their rare frequency or absence in control populations [7, 14, 15] (Table 1). Subsequent ES studies identified seven pathogenic *ARHGAP29* variants in seven unrelated families. These include one nonsense variant, four splice site alterations and two missense variants (p.Arg872Val; p.Ser552Pro) [12, 17–19] (Table 1). The clinical phenotype varied from microform cleft lip (CL) to complete bilateral cleft lip and palate (CLP). Functional studies showed that in zebrafish embryos expressing the Arhgap variant Ser552Pro, protein activity decreased, and keratinocyte migration was slowed compared to wild-type [17]. In addition, *Arhgap29* K326X mouse embryos exhibited abnormal oral adhesion during orofacial development [11].

**Table 1:** Rare ARHGAP29 variants predicted to be likely damaging in previous publications.

HGVS	ACMG in ClinVar/Varsome	Population of study	Inheritance	Phenotype	Method of sequencing	Penetrance	Functional study	Ref.
c.2615C>T: p.Ala872Val	N.R**/**VUS	10 Chinese families	AD	NSCLP	ES	80%	None	[11]
c.1654T>C: p.Ser552Pro	N.R**/**VUS	Chinese	AD	CP	ES	87%	Cell-based scratch assay Zebrafish Western Blotting	[16]
c.698-1G>C c.2109+1G>A c.1576+1G>A c.1475C>A: p.Ser492*	P/P P/P N.R/LP P/P	173 Brazilian probands and 15 from UK	AD	CL NSCLP	ES	59%	None	[17]
c.1920+1G>A	P**/**P	Chinese	AD	CL NSCLP	ES	100%	mRNA study	[18]
c.62_63delCT: p.S21Yfs*20 c.976A>T: p.Lys326* c.1865C>T: p.Thr622Met c.2533A>G: p.Ile845Val	N.R/VUS P/LP VUS/LB N.R/LB	872 cases and 802 controls US and Philippines	AD	CL NSCLP	Sequencing of nine exon (GWAS candidate region)	N.A	None	[7]
c.1939C>T: p.Arg647* c.2367G>A: p.Trp789* c.3118G>T: p.Gly1040*	N.R/LP N.R/LP N.R/VUS	1,409 Asian and European trios	AD	NSCLP	Sequencing of GWAS candidate regions	N.A	None	[14]
c. 94A>T: p. Lys32*	N.R/LP	60 Indian patients and 60 healthy control	AD	NSCLP	Sequencing of exon 1 (GWAS candidate region)	N.A	None	[13]

*N.R* not reported in ClinVar, *VUS* variants of unknown significance, *P* pathogenic, *LP* likely pathogenic, *LB* likely benign, *AD* autosomal dominant, *N.A* not available, *ES* exome sequencing, *CL* cleft lip, *NSCLP* Non-syndromic cleft lip and palate, *CP* cleft palate.

Here, we report the results of a genetic screen of a large series of NsOFC patients for *ARHGAP29* variants using ES and bioinformatic analyses.

## Subjects and methods

### Samples

Clinical information and blood DNA samples were obtained from NsOFC patients and their relatives (when available, the patient and both parents (trios), and sometimes from extended family members, such as grandparents, uncles, aunts, and cousins) at the Centre Labio-Palatin, Cliniques Universitaires St Luc, Brussels, Belgium; Amiens-Picardie Hospital, France; and University of Campinas in Brazil. Prior to enrollment into the study, participants were informed about study design and objectives. Participants signed an informed consent that was approved by the institutional review boards. A standardized questionnaire was filled out for each participant, and the referring physician assessed the family history and clinical phenotype. The study procedure was endorsed by the ethical committee of the medical faculty at University of Louvain, Brussels, Belgium (2016/10 OCT/438 – BE403201629786). For exome sequencing, we selected available samples from affected individuals and their parents. The study population included individuals who had received (a) standard genetic test(s), involving normal karyotype, and/or tested negative for 22q11.2 deletion (MIM:188400), Interferon regulatory factor 6 (*IRF6*), or grainyhead like transcription factor 3 (*GRHL3*) mutations. Patients without prior genetic testing were also included. Blood DNA samples (*n* = 335) were selected from 224 unrelated families (97 familial cases and 127 sporadic) including 224 probands and 111 their affected/unaffected relatives. Additional blood samples were collected for co-segregation studies for families with an identified putative pathogenic variant in *ARHGAP29*.

### Exome sequencing

DNAs were extracted from blood samples drawn from patients using Wizard Genomic kit (Promega), and DNA concentrations were measured with a Nanodrop 8000 (Thermo Scientific) and Qubit 2.0 (Thermo Fisher Scientific). ES was performed by Macrogen on Illumina HiSeq or NovaSeq machines, using either Agilent SureSelect (v6 or v7) or Twist Human-core Exome Capture kits. Raw data (.fasta files) were aligned to the reference human genome assembly (GRCh38) using BWA 0.7.15 (Li and Durbin, Bioinformatics 2009). Aligned sequences (.bam files) were processed using Samtools 1.12 “MarkDup” (for marking duplicates) and GATK 4.2 “BQSR” (for base quality scores recalibration). Variant calling was then performed using GATK 4.2 “Haplotype Caller” (following Broad Institute best practices). The variant call (.vcf) files generated were annotated, imported and further analyzed on Highlander 17.18 (https://sites.uclouvain.be/highlander/), the in-house bioinformatics framework of the Genomics and Bioinformatics platform of UCLouvain (PGEN: https://www.deduveinstitute.be/pgen-bioinformatics), with a local database currently containing more than 4300 ES categorized by pathology. Highlander provides extensive variant-annotation, filtering and visualization [20].

### Variant Filtering

Filtering was carried out on the 335 DNA samples (224 probands and 111 their affected and/or unaffected relatives) using Highlander [20].

Filtering was retained for variants that satisfied the following criteria:

(i) pass GATK standard quality-control filters; (ii) within a list of 544 candidate genes for oral clefts; (iii) <1% allele frequency in the population with the maximum allele frequency in GnomAD ES samples (https://gnomad.broadinstitute.org/help/popmax), Regeneron Genetics Center (RGC) and deCODE Allele Frequency (deCAF) databases ; (iv) present in less than three different pathologies in the in-house database of 3621 germline ES among 51 various pathologies; (v) Missense variants were retained only if a minimum 8 out of 20 tools indicated a deleterious effects (DAMAGING in Mutation Taster, FATHMM, FATHMM-XF, Polyphen2 (HDIV), Provean, SIFT4G, Mutation Assessor, MCAP, LRT, Lists2, Deogen, ClinPred, BayesDel (with MaxMAF), PrimateAl and MetaSVM, or a score >20 in CADD phred, >0.5 in VEST, >0.5 in REVEL, >0.75 in MVP, >0.75 in MutPred), calculated as a “consensus prediction”; (vi) Changes affecting splicing: splice site changing variants were retained by consensus prediction +300, plus the number of the above-mentioned tools predicting pathogenicity; (vii) Frameshift and nonsense: Three of the 20 algorithms have the capability to predict the pathogenicity of frameshift mutations. Our consensus prediction approach assigned a score of +400 for frameshift and stop-gained variants, plus number of the above-mentioned tools predicting pathogenicity [20].

ACMG guidelines including 12 criteria such as computational predictions, variant frequency (PM2), clinical and functional evidence (PVS1: Pathogenic Very Strong) were applied and considered to interpret pathogenicity. The common classification of variants into different categories (e.g., pathogenic, likely pathogenic, VUS, benign, likely benign) were used, and benign and likely benign variants were excluded [21]. Varsome and Franklin were used to interpret pathogenicity status based on ACMG guidelines [22, 23].

### Segregation

Segregation analysis of the identified variants was carried out on both affected and/or unaffected family members. This was performed by Sanger sequencing on an ABI 3130XL genetic analyzer (Applied Biosystems) for the three members of CLP-1251 (father, mother and grandfather) and two members of CLP-860 (brother and grandfather), while the parents of CLP-1309 and CLP-1399, and father of CLP-860 were analyzed using ES.

### Prediction tools for translation initiation sites

Two prediction tools, PreTIS and NetStart, were used to identify alternative translation initiation sites and corresponding open reading frames (ORFs). PreTIS, a web-based service, predicts non-canonical translational start sites within the 5’ untranslated region (UTR) of mRNA sequences [24]. NetStart employs neural networks to determine translation start sites in vertebrate and Arabidopsis thaliana nucleotide sequences [25].

## Results

**Fig. 1:**
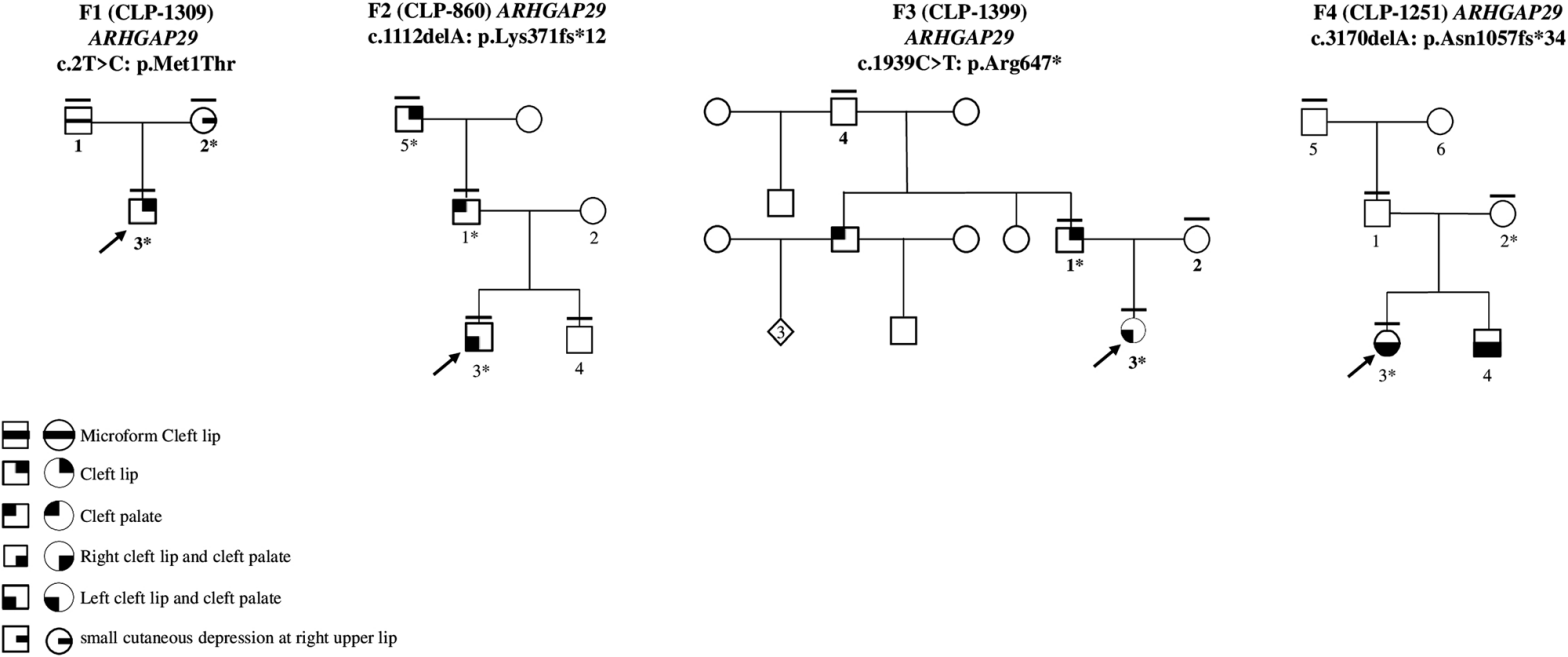
Family number; mutated gene and variant, above the pedigree. Clinically studied individuals, horizontal bar; blood sample available, numbered individual; *, variant carrier; phenotypes specified by symbols.

We identified four putative pathogenic variants in *ARHGAP29* (ENST00000260526, NM_004815.4) in four multiplex families. In family 1, a proband with unilateral CL and his mother with small cutaneous depression at right upper lip were found to have a missense variant concerning the initiation methionine (c.2T>C, Met1Thr). In family F2, the proband with complete unilateral left CLP was identified to have a variant shifting the reading frame (c.1112delA, Lys371Serfs*12). This was also present in the affected father with left CP and his grandfather with CL. In family F3, the proband with a complete left CLP and her father with minimal CL had a nonsense variant (c.1939C>T, p.Arg647*). In family F4, the proband with complete bilateral CLP and her unaffected mother had a variant which shifts the reading frame (c.3170delA, Asn1057fs34*) (Fig. 1).

### Family 1

The index individual (CLP-1309-3) exhibited unilateral CL (Fig. 1A). Father has a microform of CL and clinical examination of his mother was practically unremarkable. However, a mild microform could not be excluded as she presents a small cutaneous depression at right upper lip with normal ultrasound of orbicularis oris muscle. ES in proband and his mother unraveled a heterozygous nucleotide substitution (c.2T>C) on exon 1 of *ARHGAP29* with a consensus score of 11/20 (Fig. 1A) [20].

This nucleotide change was reported three times in gnomAD (*N* = 152,090, alternative allele frequency <0.0001), 27 times in RGC (*N* = 985,196, alternative allele frequency <0.0001) and once in deCAF (*N* = 276,318, alternative allele frequency <0.00001) databases, whereas it was not reported in our local database or in LOVD [20, 26]. The variant is predicted to cause the substitution of the first methionine by threonine (p.Met1Thr; NP_001315596.1). The Combined Annotation Dependent Depletion (CADD) score was 24.2 [27]. In the Geno2MP database, the same variant was reported in one individual with nephrotic syndrome and short stature (details not provided) [28]. In addition, the variant (c.1A>G: p.(Met1Val), not the same nucleotide variant) was reported in two individuals with malformation of the heart and great vessels, and abnormality of hindbrain morphology [28]. This change, substituting the first methionine with valine, is also documented in ClinVar without specific information [29].

**Fig. 2:**
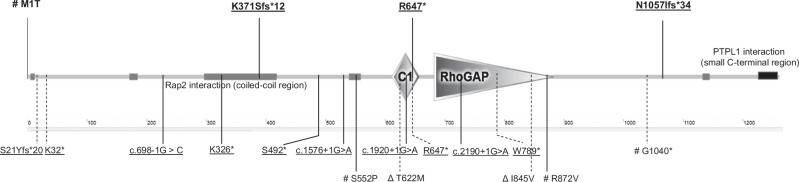
ARHGAP29 protein domains with variants: our findings above the figure; reported ones below the figure; dashed line, rare variants discovered through direct sequencing on candidate regions from GWAS studies; continuous lines, rare variants found by exome sequencing. Symbols for ACMG classification of variants in Varsome: Δ, likely benign; #, VUS, and underlined variants, pathogenic/likely pathogenic.

The next methionine is located 28 amino acids downstream. Therefore, due to this substitution, the encoded *ARHGAP29* is expected to lose its function (Fig. 2) [24, 25]. PreTIS analysis revealed optional start codons with high and very high confidence level. They would lead to the generation of shorter ORFs of varying lengths, including 15, 30, 111, 138, and 3801 nucleotides. The highest confidence start codon would result in an ORF length of only 15 nucleotides [24]. NetStart’s predictions indicated 15 potential start points within the cDNA sequence, exhibiting high scores. These start points range from nucleotide 268 to 3536 in the cDNA sequence [25]. Following the ACMG guidelines, the variant was assessed as having a supporting and moderate pathogenic impact based on the PVS1 and PM2 criteria, respectively. Therefore, ACMG classification of this variant is VUS [21–23].

### Family 2

The proband (CLP-860-3) had a complete unilateral left CLP. His father and grandfather had left CP and CL, respectively. Clinical examination of his brother and mother was unremarkable. ES results in the proband and his father unraveled a heterozygous nucleotide deletion (c.1112delA) on exon 11 of *ARHGAP29* with a consensus score of 403. Co-segregation study confirmed that the affected paternal grandfather was a carrier, while mother and brother were unaffected and non-carrier (Fig. 1B).

This variant was not reported in gnomAD, RGC, or deCAF databases, nor was it observed in the local database [20]. It was not reported in ClinVar, LOVD or Geno2MP [26, 28, 29]. The deletion is predicted to cause a frameshift [p.(Lys371Serfs*12); NP_001315596.1] and it is likely to undergo NMD. If not, it would encode a truncated protein expected to lose the C1, RhoGAP and *PTPL1-*interaction domains (Fig. 2). According to ACMG guidelines, the variant was assessed as having a very strong pathogenic impact based on the PVS1 criterion and moderate pathogenic impact according to PM2 criterion, and it is categorized as pathogenic [21–23].

### Family 3

The proband (CLP-1399-3) had a complete left CLP and her father had a minimal CL. Her paternal uncle had CP, whereas his four children did not have any signs of clefting (Fig. 1C). Clinical examination of her mother was unremarkable.

A trio analysis using ES revealed a heterozygous nucleotide change (c.1939C>T) on exon 18 of *ARHGAP29* with a consensus score of 403 on the index patient and her father (Fig. 1C). Paternal uncle with CP was not tested for this variant. The variant was not present in gnomAD, RGC, deCAF, or local databases [20]. It was not observed in ClinVar, LOVD or Geno2MP [26, 28, 29]. In an Asian cohort study, the same variant was reported in the direct sequencing of candidate regions identified in GWAS studies [15]. The CADD score (ranging from 1 to 99, likely pathologic considered usually more than 20) is 42 for this variant [27]. This change is predicted to cause a nonsense mutation (p.(Arg647*); NP_001315596.1) resulting in NMD or a truncated protein. If produced, this nonsense mutation in the C1 domain is expected to lose the RhoGAP domain and *PTPL1*-intaraction domain (Fig. 2).

According to ACMG guidelines, the variant was assessed as having a very strong and moderate pathogenic impact based on the PVS1 and PM2 criteria, respectively. Moreover, the variant is classified as a supporting PP3 criterion due to multiple lines of computational evidence supporting a deleterious effect on the gene or gene product (conservation, evolutionary, splicing impact, etc.) [21]. Thus, the variant was categorized as likely pathogenic [22, 23].

### Family 4

The index individual (CLP-1251-3) and her brother had a complete bilateral CLP. The parents’ clinical examination was unremarkable. ES was performed on proband, revealing a heterozygous nucleotide deletion (c.3170delA) in exon 23 of *ARHGAP29* with a consensus score of 403. Co-segregation analysis demonstrated that the unaffected mother was a carrier, whereas the unaffected father, paternal grandmother and grandfather were non-carriers (Fig. 1.F4). DNA blood sample of her affected brother was not available for verification of the presence of the variant (Fig. 1A). The variant has not been reported in gnomAD, RGC or deCAF databases. This variant was not observed in the local database either [20], nor is it reported in ClinVar, LOVD or Geno2MP [26, 28, 29].

This one nucleotide deletion leads to a frameshift (p.(Asn1057Ilefs34*); NP_001315596.1) with a premature termination codon (PTC) 34 amino acids downstream [20]. The gene is intolerant for LoF mutations, as its LoF intolerance value (PLi) is 1.00, the maximum [30]. The variant is located in the last exon, thus the mRNA is less likely to undergo nonsense mediated mRNA decay (NMD), but rather likely to encode a truncated protein. Therefore, it is predicted to lose the small C-terminal domain for interaction with Protein Tyrosine Phosphatase-Like 1 (*PTPL1)*.

Following the ACMG guidelines, the variant was categorized as PVS1:strong (Pathogenic Very Strong) owing to its predicted impact leading to protein loss of function. Additionally, the variant was classified as PM2 (Moderate) due to allele frequency in the general population databases [21]. In Varsome and Franklin databases, this variant is classified as likely pathogenic [22, 23].

## Discussion

To date, eight single nucleotide variants, which are considered likely to be pathogenic based on predictive algorithm(s) and rare allele frequencies or absence in control populations, have been reported in *ARHGAP29* in NsOFC patients by sequencing of GWAS candidate regions [7, 13, 14] (Table 1, Fig. 2). However, categories of ACMG in Varsome for these eight variants were: 4 likely pathogenic, 2 VUS and 2 likely benign. In addition, ES studies have identified seven variants in *ARHGAP29* (5 pathogenic and 2 VUS based on ACMG) in seven unrelated families: one nonsense (p.Ser492*) mutation, four splice site changes (c.698-1G>C; c.2109+1G>A; c.1576+1G>A; c.1920+1G>A) and two missense variants (p.Arg872Val; p.Ser552Pro) [12, 17–19] (Table 1, Fig. 2). We identified four novel putative pathogenic variants (1 pathogenic, 2 likely pathogenic and 1 VUS) in four NsOFC probands, including one replacement of the first methionine, one nonsense variant, and two heterozygous frameshifts, increasing the total to 19 rare *ARHGAP29* variants (6 pathogenic, 6 likely pathogenic, 5 VUS and 2 likely benign based on the ACMG classifications in Varsome). This underscores the strong involvement of *ARHGAP29* in NsOFC pathogenesis.

In Family 1, we identified a variant that does not segregate with the phenotype. The affected proband has the variant, as does the unaffected mother. However, the father, who has a minimal cleft lip does not carry the variant. This suggests that the identified variant may not be causative in this family, and that other genetic and/or environmental factors contribute to the phenotype, highlighting the complexity of NsOFC inheritance.

In protein synthesis, the initial methionine is crucial; alterations or deletions can disrupt translation or folding. Alterations in this region, such as the c.Met1Thr variant observed in *ARHGAP29* in family 1, can lead to the complete absence of the protein or its production in a truncated form. Our PreTIS analysis highlighted the existence of varied, shorter ORFs, with lengths ranging from 15 to 3801 nucleotides, the strongest one associated with the smallest ORF of only 15 nucleotides in length [24]. The variation in potential start sites, as seen in *ARHGAP29’*s c.Met1Thr variants, indicates a risk of losing different protein domains and structure, underscoring the need for precise start site prediction to understand protein structure and function in genetic mutations [31–35].

The four putative pathogenic variants showed an 89% penetrance across our four families, underscoring a strong genetic influence of *ARHGAP29* variants for NsOFC (one out of 9 was unaffected, 8 out of 9 were affected). The penetrance of *ARHGAP29* variants was calculated to range from 59% to 100% in the previous reports, underscoring a moderate to high penetrance. Incomplete penetrance in cleft lip and palate (CLP) exemplifies the phenomenon where individuals carrying mutations in specific genes, such as *IRF6, MSX1*, and *TP63*, do not consistently express the associated phenotype. This variability can be attributed to complex interactions between genetic background, environmental influences, and epigenetic modifications that modulate gene expression and developmental processes [2, 6, 9, 12, 17–19]. Given the small number of families with *ARHGAP29* variants, further studies are needed to determine if there is a significant difference in penetrance between males and females [12, 17–19].

The phenotypes varied from microform CL to complete bilateral CLP and CP only, without other craniofacial defects, and could therefore be added to the list of genes involved in all types of clefts such as *IRF6, GRHL3*, *TP63* and *COL2A1*. To the best of our knowledge, microform CL has not been reported yet. Moreover, intrafamilial clinical variability (CL, CLP and CP) was observed in two families with *ARHGAP29* frameshift and nonsense variants. However, in family 3, the paternal uncle with CP was not available for *ARHGAP29* variant testing. This is similar to reports of other *ARHGAP29* variants, where the clinical presentation varied widely within the same family [18, 19]. In addition, this intrafamilial clinical variability is also seen e.g. in *IRF6* mutated cleft patients, whose syndromic signs can be limited to inconspicuous lip pits and/or synechia, or be completely absent [36]. Therefore, it is likely that genetic or environmental modifiers play an important role even in the Mendelian forms of NsOFC development. No syndrome caused by germline *ARHGAP29* mutations has so far been reported. This is in contrast to several other NsOFC genes as causative likely pathogenic variants can be identified in about 10% of NsOFC cases in the genes causing SyOFC [6, 36, 37]. *ARHGAP29* should thus be included in diagnostic genetic testing for NsOFC, as it may be mutated in 4% of familial cases (4/97 in our cohort) with high penetrance. The most frequent cleft type seems to be cleft lip with palate (CLP).

The variants are predicted to result in either *ARHGAP29* haploinsufficiency by degradation of mutated mRNA by NMD, and/or to loss of function due to the lack of important functional domains, such as the RhoGAP, Rap2 interaction (coiled-coil region), C1, RhoGAP and PTPL1-interaction domains (Fig. 2). One variant is located in the last exon, thus it is less likely to undergo nonsense mediated mRNA decay (NMD), but rather to encode a truncated protein lacking the *PTPL1* interacting domain (Fig. 2). PTPL1, a non-receptor tyrosine phosphatase, forms a complex with ARHGAP29 through its PDZ domain, particularly in linking membrane proteins to the cytoskeleton and constructing signaling complexes [38]. This interaction potentially prevents RhoA activity, regulating RhoA-LIMK-cofilin pathway, which is significant in cytoskeletal dynamics. Moreover, this interaction is crucial in regulating cell growth, survival, and proliferation via PI3K/AKT/mTOR signaling pathway [39]. Therefore, these pathways are essential in developmental processes and their dysregulation in either of these pathways can potentially disrupt the normal development of craniofacial structures, leading to conditions like cleft lip and palate [40].

In summary, the finding of these variants and the predicted loss of important functional domains such as the RhoGap domain in *ARHGAP29* increases the growing evidence implicating abnormal Rho GTPase signaling in the pathogenesis of orofacial clefts. These results extend the mutational spectrum of *ARHGAP29* and demonstrate that it is an important genetic factor underlying variable NsOFC phenotypes. *ARHGAP29* should be included in diagnostic genetic testing for NsOFC, especially for familial cases, as it may be mutated in ∼4% of them (4/97 in our cohort) with high penetrance (89%).

## Data Availability

All data generated or analyzed during this study are included in the published article. The four variants have been submitted to the Leiden Open Variation Database (LOVD) under the gene-specific database for *ARHGAP29* and are accessible via the following links: [https://databases.lovd.nl/shared/variants/0001011838#00002793; https://databases.lovd.nl/shared/variants/0001011773#00002793; https://databases.lovd.nl/shared/variants/0001011774#00002793; https://databases.lovd.nl/shared/variants/0001011775#00002793], with submission numbers [Variant #0001011838, Variant #0001011773, Variant #0001011774, and Variant #0001011775].
